# Progressive dysexecutive syndrome due to Alzheimer’s disease: a description of 55 cases and comparison to other phenotypes

**DOI:** 10.1093/braincomms/fcaa068

**Published:** 2020-05-27

**Authors:** Ryan A Townley, Jonathan Graff-Radford, William G Mantyh, Hugo Botha, Angelina J Polsinelli, Scott A Przybelski, Mary M Machulda, Ahmed T Makhlouf, Matthew L Senjem, Melissa E Murray, Ross R Reichard, Rodolfo Savica, Bradley F Boeve, Daniel A Drubach, Keith A Josephs, David S Knopman, Val J Lowe, Clifford R Jack, Ronald C Petersen, David T Jones

**Affiliations:** f1 Department of Neurology Mayo Clinic, Rochester, MN 55902, USA; f2 Department of Psychiatry and Psychology, Mayo Clinic, Rochester, MN 55902, USA; f3 Department of Biomedical Statistics, Mayo Clinic, Rochester, MN 55902, USA; f4 Department of Diagnostic Radiology, Mayo Clinic, Rochester, MN 55902, USA; f5 Department of Molecular Neuroscience, Mayo Clinic, Jacksonville, FL 32224, USA; f6 Department of Laboratory Medicine and Pathology, Mayo Clinic, Rochester, MN 55902, USA

**Keywords:** dysexecutive, early-onset Alzheimer’s disease, CSF biomarkers, FDG-PET, tau PET

## Abstract

We report a group of patients presenting with a progressive dementia syndrome characterized by predominant dysfunction in core executive functions, relatively young age of onset and positive biomarkers for Alzheimer’s pathophysiology. Atypical frontal, dysexecutive/behavioural variants and early-onset variants of Alzheimer’s disease have been previously reported, but no diagnostic criteria exist for a progressive dysexecutive syndrome. In this retrospective review, we report on 55 participants diagnosed with a clinically defined progressive dysexecutive syndrome with ^18^F-fluorodeoxyglucose-positron emission tomography and Alzheimer’s disease biomarkers available. Sixty-two per cent of participants were female with a mean of 15.2 years of education. The mean age of reported symptom onset was 53.8 years while the mean age at diagnosis was 57.2 years. Participants and informants commonly referred to initial cognitive symptoms as ‘memory problems’ but upon further inquiry described problems with core executive functions of working memory, cognitive flexibility and cognitive inhibitory control. Multi-domain cognitive impairment was evident in neuropsychological testing with executive dysfunction most consistently affected. The frontal and parietal regions which overlap with working memory networks consistently demonstrated hypometabolism on positron emission tomography. Genetic testing for autosomal dominant genes was negative in all eight participants tested and at least one *APOE* ε*4* allele was present in 14/26 participants tested. EEG was abnormal in 14/17 cases with 13 described as diffuse slowing. Furthermore, CSF or neuroimaging biomarkers were consistent with Alzheimer’s disease pathophysiology, although CSF p-tau was normal in 24% of cases. Fifteen of the executive predominate participants enrolled in research neuroimaging protocols and were compared to amnestic (*n* = 110), visual (*n* = 18) and language (*n* = 7) predominate clinical phenotypes of Alzheimer’s disease. This revealed a consistent pattern of hypometabolism in parieto-frontal brain regions supporting executive functions with relative sparing of the medial temporal lobe (versus amnestic phenotype), occipital (versus visual phenotype) and left temporal (versus language phenotype). We propose that this progressive dysexecutive syndrome should be recognized as a distinct clinical phenotype disambiguated from behavioural presentations and not linked specifically to the frontal lobe or a particular anatomic substrate without further study. This clinical presentation can be due to Alzheimer’s disease but is likely not specific for any single aetiology. Diagnostic criteria are proposed to facilitate additional research into this understudied clinical presentation.

## Teaser

We present a retrospective case series of 55 individuals presenting with a progressive dysexecutive syndrome with biomarker evidence of Alzheimer’s disease. This clinical syndrome should be recognized as a distinct clinical phenotype that is disambiguated form behavioural presentations and not linked to a particular anatomic substrate without further study.

## Graphical Abstract

**Graphical Abstract:**
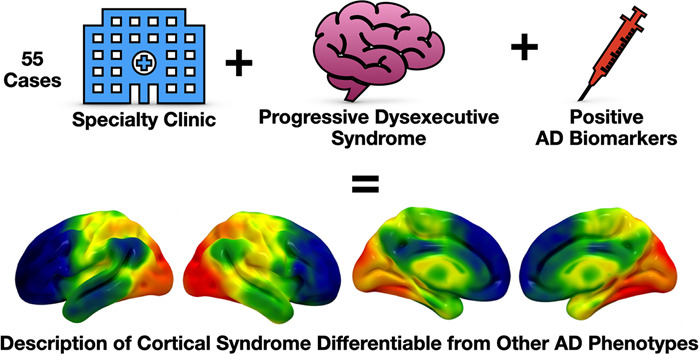

## Introduction

Emerging out of clinical consultations in a tertiary behavioural neurology clinic, we recognized a relatively common pattern among patients presenting with a progressive dementia syndrome characterized by predominant executive dysfunction, early age of onset and positive biomarkers for Alzheimer’s pathophysiology. These patients did not present amnestically like the typical Alzheimer’s disease clinical syndrome. Given the lack of diagnostic criteria and a paucity of published literature describing the characteristics of these patients, they have a prolonged period before diagnosis and are frequently misdiagnosed.

Evidence for a ‘frontal phenotype’ of Alzheimer’s disease has accumulated since the initial reports of a case series (*N* = 3) of a subgroup of patients with pathologically confirmed Alzheimer’s disease and disproportionate impairment on executive cognitive tests (Trail Making Test A and letter fluency) (Johnson *et al.*, 1999). However, there has been ambiguity in case definitions conflating syndromes (executive versus behavioural) and anatomy (frontal lobe). The International Working Group 2 described a frontal phenotype of Alzheimer’s disease as a progressive behavioural syndrome in the setting of positive Alzheimer’s disease biomarkers that may occur alongside cognitive impairment in executive function (Dubois *et al.*, 2014). In an aim to better represent the clinical and neuroimaging features of such cases, a behavioural/dysexecutive variant of Alzheimer’s disease was then proposed (Ossenkoppele *et al.*, 2015). In this study, Ossenkoppele *et al.* described that the dysexecutive variant presented with cognitive symptoms 83% of the time and behavioural symptoms 3% of the time, and a behavioural variant presented with cognitive symptoms 53% of the time and behavioural symptoms 25% of the time (12% met criteria for both). The imaging characteristics of these participants included atrophy in the temporoparietal cortex and precuneus, with only subtle frontal lobe atrophy (Ossenkoppele *et al.*, 2015). Whether these two variants represent a clinical spectrum or two distinct variants of Alzheimer’s disease requires further study. This study operationalized a dysexecutive Alzheimer’s disease syndrome using a composite score of executive functions (Digit Span backwards, Trail Making Test Part B, Stroop Color-Word Test and Letter Fluency) being relatively more impaired than a composite score of memory function (Delayed Recall of the Californian Verbal Learning Test or Dutch version of the Rey Auditory Verbal Learning Test and Benson Figure Test or the Visual Association Test) in individuals who had an autopsy and/or biomarker confirmation of Alzheimer’s disease pathology.

In the current study, we aim to provide an operational definition of a progressive dysexecutive syndrome that can be used to describe a clinical presentation to memory clinics that is free from an anatomic description or an underlying aetiological association (e.g. Alzheimer’s disease), as well as free from requirements of particular neuropsychological test results to facilitate further study of this population in a routine clinical context. Neuropsychological testing requirements are not included in the criteria for several reasons: patients may be too impaired at the time of the clinical evaluation to be tested; there exists a lot of variability in which neuropsychological tests are administered, the availability of normative data for younger individuals are lacking as these are often designed to assess cognitive performance in older age individuals; and the fact that most neuropsychological tests require some element of core executive function for performance, resulting in a ‘multi-domain’ pattern of dysfunction when core executive functions are impaired. This is in line with current criteria in the field that do not specify which cognitive test must be impaired to meet the criteria (McKhann *et al.*, 2011; Dubois *et al.*, 2014).

While it is our experience that many cases presenting with a progressive dysexecutive syndrome eventually are determined to have Alzheimer’s disease as the underlying aetiology, important other causes have been observed in our clinic (e.g. dementia with Lewy bodies, frontotemporal lobar degeneration, Creutzfeldt-Jakob disease and others). However, to raise awareness of the predominant aetiology of the progressive dysexecutive syndrome found in our clinic and to contextualize this syndrome within the broader Alzheimer’s disease literature, we report here only on cases that were on the Alzheimer’s disease continuum based on the new research framework (Jack *et al.*, 2018).

We present an initial description of the clinical presentation, neuropsychological profiles, EEG patterns, genetic results, neuropathologic findings and multimodal neuroimaging characteristics of 55 cases that presented to our clinic with a progressive dysexecutive syndrome that had biomarker or neuropathologic evidence of Alzheimer’s disease. We conclude by discussing these findings to inform aetiologically agnostic diagnostic criteria for a progressive dysexecutive syndrome with added aetiologic parameters for Alzheimer’s disease (dysexecutive Alzheimer’s disease) to facilitate additional research into this poorly understood clinical presentation in memory clinics.

## Materials and methods

### Patient consent

Design and implementation of this single-centre retrospective study met HIPAA guidelines and was approved by the Mayo Clinic Institutional Review Board. Participants signed a research document at their first clinical visit to have their data used in research. Informed consent was obtained for clinical studies [e.g. lumbar puncture and ^18^F-fluorodeoxyglucose (FDG)–PET] and subsequent research studies (e.g. tau and amyloid PET).

### Participants, clinical features and diagnosis

A series of participants presenting to an outpatient behavioural neurology clinical practice with a progressive dysexecutive syndrome were collected at Mayo Clinic Rochester between March 2014 and July 2017. A retrospective review of these participants’ clinical Electronic Medical Record was then performed to include those who met the provisional diagnostic criteria for possible or definite dysexecutive Alzheimer’s disease (Box 1) via consensus opinion from four subspecialty trained behavioural neurologists (R.A.T., J.G.-R., H.B. and D.T.J.). This was a modified version of the International Working Group 2 criteria for the frontal phenotype of atypical Alzheimer’s disease (Dubois *et al.*, 2014) and the current NIA-AA research criteria framework (Jack *et al.*, 2018).

One participant did not have *in vivo* biomarkers but had pathology proven Alzheimer’s disease and was also included. Each participant was seen and diagnosed after a visit with a behavioural neurologist. The clinical history and examination were not standardized and were at the discretion of the behavioural neurologist. Behavioural symptoms were probed and documented as positive or negative in the clinical history. Further testing ordered (including formal neuropsychological, EEG and genetic testing) also varied across participants and was based on patient–clinician shared decision-making. Amyloid PET and tau PET were obtained for participants who were also enrolled in our Alzheimer’s Disease Research Center (ADRC).

Exam findings of ideomotor apraxia (e.g. ‘show me how you would hammer a nail’), Luria’s motor series (Luria, 1966) and aphasia (using a standard language screen consisting of auditory comprehension, reading comprehension, naming, repetition, narrative picture description and writing) were included in the analysis if a patient’s chart explicitly mentioned the results of these tests. In six cases, participants were referred to a speech pathologist at our institution for detailed speech and language evaluation. Simultanagnosia testing was performed using a combination of Navon figures (Navon, 1977), Ishihara plates (Brazis *et al.*, 1998) and an overlapping figure with five items (Riddoch and Humphreys, 1993). All participants underwent bedside cognitive screening in the form of the Kokmen Short Test of Mental Status (Kokmen *et al.*, 1991).

### Neuropsychological testing

Formal neuropsychological testing was performed in 32 out of 55 participants. Neuropsychological testing was not performed in 23 participants due to either travel logistics or severity of cognitive impairment on bedside screening. The neuropsychological battery differed slightly across participants depending on whether they were enrolled as a research participant in the ADRC or undergoing a standard clinical evaluation. The neuropsychological battery for each participant included combinations of the following tests.

Dementia Rating Scale-2 (DRS-2) (Jurica *et al.*, 2001), Rey Auditory Verbal Learning Test (AVLT) (Rey, 1964), Wechsler Memory Scale-Revised (WMS-R) or 3rd Edition (WMS-III) Logical Memory (LM) I and II and Visual Reproductions (VR) I and II subtests (Wechsler, 1987, 1997), Wechsler Adult Intelligence Scale-Revised (WAIS-R) or 3rd Edition (WAIS-III)—Digit Span (DS) and Letter Number Sequencing (LNS) subtests (Wechsler, 1981, 1997), Rey-Osterrieth Complex Figure copy (Osterrieth, 1944), Trail Making Test Part A (Trails A) (Reitan, 1958; Spreen and Strauss, 1998), Trail Making Test B (Spreen and Strauss, 1998), Stroop Test: Word-Reading, Color-Naming and Interference trials (Stroop, 1935), Boston Naming Test (BNT) (Kaplan *et al.*, 1976), Controlled Oral Word Association Test (COWAT) (Ruff *et al.*, 1996) and Category Fluency (Lucas *et al.*, 1998).

Raw scores were converted to age-adjusted standard scores. Mayo Older Americans Normative Studies (MOANS) were used for all tests (Petersen *et al.*, 1992; Ivnik *et al.*, 1997; Lucas *et al.*, 1998; Steinberg *et al.*, 2005; Machulda *et al.*, 2007) with the exception of WMS-III and WAIS-III subtests for which MOANS are not available. For these subtests, published age-adjusted norms from their respective manuals were used. There are three important caveats to these data. First, MOANS norms only extend down to age 56 and four participants were younger than this. In these cases, the youngest MOANS age bracket (56–60) was used to derive normative scores. Second, given the small sample, WAIS-R and WAIS-III scaled scores and WMS-R and WMS-III scaled scores were combined across participants. Finally, two participants had prior neuropsychological evaluations and the data included here reflect their second evaluation (i.e. the evaluations in closest proximity to the imaging and biomarkers). All MOANS and standard scores were converted to z-scores for data presentation.

### CSF biomarkers

Lumbar puncture for CSF testing occurred during the week of the initial clinical visit. For Alzheimer’s disease biomarker testing, 2 ml of CSF were collected from each participant at Mayo Clinic, transferred into polypropylene transfer tubes, frozen at −85°C and transported overnight to Athena Diagnostics (Worcester, MA). An enzyme-linked immunosorbent assay employed by Athena Diagnostics was used for CSF analysis for all participants in the present study. An Aβ42: Tau Index <1 is used by Athena and has a sensitivity of 85–94% and a specificity of 83–89% in differentiating clinically diagnosed Alzheimer’s disease from non-Alzheimer’s disease conditions such as vascular dementia and frontotemporal dementia (Hulstaert *et al.*, 1999; Andreasen *et al.*, 2001).

### NeuroImaging

FDG–PET images were acquired using a PET/CT scanner (GE Healthcare) operating in 3D mode. Participants were injected in a dimly lit room with FDG, and after a 30-min uptake period, an 8-min FDG scan was performed, which consisted of four 2-min dynamic frames following a low-dose CT transmission scan. Standard acquisition and vendor reconstruction parameters were used. FDG–PET scans were processed using CortexID software (GE Healthcare). The activity in each participant’s PET dataset was normalized to the pons and compared with an age-segmented normative database, yielding z-score 3D-stereotactic surface projection images.

Amyloid-PET imaging was done with Pittsburgh compound B, synthesized on-site with precursor purchased from ABX Biochemical Compounds. Tau PET was carried out with flortaucipir (^18^F-AV-1451), synthesized on-site with precursor supplied by Avid Radiopharmaceuticals. Image processing methods have been described previously (Jack *et al.*, 2012, 2017). Amyloid and tau PET images were scaled using a cerebellar crus grey matter region of interest (ROI), resulting in standard uptake value ratio (SUVR) images. Previously validated Meta ROIs were used to derive a single value summary measure of amyloid and tau uptake. Positive amyloid PET and tau PET SUVR cut-offs were defined as >1.42 and >1.23, respectively (Jack *et al.*, 2017). Frontal and parietal ROI data were obtained using the Mayo Clinic Adult Lifespan Template (Schwarz *et al.*, 2017). R statistical software was used for exploratory analysis between age of onset, short test of mental status (STMS) and tau PET SUVR.

### Group-wise comparison across Alzheimer’s disease phenotypes enrolled in the Mayo ADRC

A subset (*n* = 15) of the participants originally seen in the clinical practice and included in this case series were enrolled in the Mayo Clinic Alzheimer’s Disease Research Center and underwent standardized MRI and FDG–PET imaging. The Mayo Clinic Rochester Alzheimer’s Disease Research Center is a longitudinal cohort study that enrols subjects from the clinical practice at Mayo Clinic in Rochester, MN. Enrolled participants are adjudicated by a consensus panel consisting of study coordinators, neuropsychologists and behavioural neurologists. These participants were compared to typical amnestic (*n* = 110), visual (*n* = 18) and language (*n* = 7) predominant phenotypes of Alzheimer’s disease dementia enrolled in the Alzheimer’s Disease Research Center that met existing criteria for typical, posterior cortical atrophy or logogenic variant of primary progressive aphasia (Gorno-Tempini *et al.*, 2011; McKhann *et al.*, 2011; Crutch *et al.*, 2017).

Research MR scanning was performed at 3 Tesla and the 3D magnetization-prepared radiofrequency pulses and rapid gradient echo sequences as previously described (Jack *et al.*, 2008). Parameters were: TR/TE/T_1_, 2300/3/900 ms; flip angle 8°, 26 cm field of view (FOV); 256 × 256 in-plane matrix with a phase FOV of 0.94, and slice thickness of 1.2 mm. These magnetization-prepared radiofrequency pulses and rapid gradient echo parameters have been held invariant since approximately 2008. Hippocampal volume was measured with FreeSurfer (https://surfer.nmr.mgh.harvard.edu/). This structural MRI was used for pre-processing FDG–PET data. Details regarding imaging procedures have been widely reported [see Jack *et al.* (2017) and references therein].

The FDG–PET image volumes of each subject were co-registered to the subject’s own T_1_-weighted MRI scan, using a 6 degree-of-freedom affine registration with mutual information cost function. Each MRI scan was then spatially normalized to an older adult template space (Vemuri *et al.*, 2008) using a unified segmentation and normalization algorithm (Ashburner and Friston, 2005) with transforms applied to co-registered FDG–PET images. These spatially normalized images were then intensity normalized to the pons and spatially smoothed with a 6 mm full-width half-maximum Gaussian kernel. These images were then entered into a voxel-wise comparison by Alzheimer’s disease phenotype using SPM12 software (https://www.fil.ion.ucl.ac.uk/spm/) running on MATLAB version R2018a (Mathworks, Natick, MA). Results were considered significant at a voxel-wise FDR corrected *P*-value of 0.05. Group-wise comparisons of variables were performed using Kruskal–Wallis one-way analysis of variance and pairwise using Wilcoxon rank sum test in R (https://www.R-project.org/) and visualized using the ggplot2 package (Wickham, 2009).

### Genetic testing

Clinical genetic blood tests included *APOE* allele status and/or three autosomal dominant genes: Amyloid Precursor Protein (APP), Presenilin-1 (PSEN1) and Presenilin-2 (PSEN2).

### Pathology

As part of a standardized dissection and sampling protocol (Mirra *et al.*, 1991), formalin-fixed and paraffin-embedded sections of the left hemisphere were taken for immunohistochemical studies. Amyloid plaques and neurofibrillary tangles were immunohistochemically evaluated using antibodies to Aβ (6F/3D; 1:20; Novocastra VectorLabs, Burlingame, CA) and phospho-tau (AT8; 1:1000; Endogen, Woburn, MA) and staged in accordance with recommendation from the National Institute of Aging-Alzheimer’s Association (NIA-AA) and Consortium to Establish a Registry for Alzheimer’s disease (CERAD) guidelines (Mirra *et al.*, 1991; Hyman *et al.*, 2012). Thal amyloid phase was performed using Aβ immunohistochemistry (Thal *et al.*, 2002) and Braak tangle stage was performed using tau immunohistochemistry (Braak and Braak, 1991). Immunohistochemical evaluation of alpha-synuclein (LB509; 1:200; Zymed, San Francisco, CA, USA) did not reveal Lewy body pathology. TDP-43 pathology was evaluated using the MC2085 antibody (rabbit polyclonal, a gift from Leonard Petrucelli, Mayo Clinic) (Zhang *et al.*, 2009). Aging-related tau astrogliopathy was reviewed on tau immunostained sections (Kovacs *et al.*, 2016).

### Data availability

Data that support the findings in this study are available from the corresponding author upon reasonable request.

## Results

### Demographics and family history

Demographics are described in Table 1. There was a slight female predominance of 62%, a mean education of 15.2 years and a mean age of onset of 53.8 years. Thirty out of 55 (55%) participants reported a first or second degree relative with a history of dementia. Only 2 out of 30 relatives reportedly had young onset dementia (<65 years old). For one of the participants with young onset family history, the participant had an *APOE* ε*4* allele present but negative autosomal dominant Alzheimer’s disease genetic testing, and the other participant had no allelic or genetic information available. At least one *APOE* ε*4* allele was present in 14/26 (54%) participants tested. Homozygous *APOE* ε*4* was present in five participants with a mean age of reported onset at 53 years, family history was present in all five (all relatives >65 years old at onset) and one participant had negative genetic testing for *APP*, *PSEN1* and *PSEN2*. Heterozygous *APOE* ε*4* was present in nine participants with a mean age of reported onset at 52.6 years, a family history in 7/9, young onset family history in one participant and negative autosomal dominant Alzheimer’s disease genetic testing in that individual. No *APOE* ε*4* allele was present in 12 participants with a mean age of reported onset at 52.8 years, a family history in 3/12, no young onset family history and 3 participants with negative autosomal dominant Alzheimer’s disease genetic testing. Overall, there were eight participants with genetic testing for *APP*, *PSEN1* and *PSEN2* and all had negative results.

**Table 1:** Broad characterization of progressive dysexecutive syndrome due to Alzheimer’s disease

Participant demographics (*N* = 55)	
Age of onset (years), mean (range)	53.8 (48–69)
Age at first visit (years), mean (range)	57.1 (48–71)
Education (years), mean (range)	15.2 (12–20)
Gender, No. (%) male	21/55 (38%)
Self-reported family history dementia, *N* (%)	30/55 (55%)
Young onset family history dementia, *N* (%)	2/55 (4%)
Genetics	No. with positive test/total tested
APP/PS1/PS2	0/8 (0%)
*APOE* ε*4* allele carrier status^a^	14/26 (54%)
Clinical and physical exam features	No. with positive finding/total number with documentation of test
Parkinsonism	2/55 (4%)
Self-reported RBD	2/55 (4%)
Ideomotor Apraxia	28/40 (70%)
Aphasia	24/31 (77%)
Simultanagnosia	11/23 (48%)
Difficulties with Luria	39/39 (100%)
Neuropsychological testing	Mean (SD)
STMS, raw score	19.9^b^ (8.9)
Dementia Rating Scale-2, raw, *N* = 32	107.1 (28.9)
Dementia Rating Scale-2, z-score, *N* = 32	−2.0 (0.9)
WAIS-R/III	
Letter Number Sequence, z-score, *N* = 14	−1.7 (0.5)
Digit Span, z-score, *N* = 20	−1.0 (0.7)
WMS-R/III	
Logical Memory I, z-score, *N* = 20	−2.3 (0.8)
Logical Memory II, z-score, *N* = 20	−1.9 (0.8)
Visual Reproduction I, z-score, *N* = 17	−2.4 (0.7)
Visual Reproduction II, z-score, *N* = 17	−1.8 (0.9)
AVLT Trial 1, z-score, *N* = 22	−1.2 (0.8)
AVLT LOT, z-score, *N* = 22	−1.4 (0.9)
AVLT DR, z-score, *N* = 22	−2.0 (0.6)
Trail Making Test A, z-score, *N* = 28^c^	−1.9 (1.1)
Trail Making Test B, z-score, *N* = 22^d^	−2.4 (0.9)
Stroop Word, z-score, *N* = 19	−1.5 (0.8)
Stroop Color, z-score, *N* = 19	−1.8 (1.0)
Stroop Interference, z-score, *N* = 19	−2.1 (0.9)
COWAT, z-score, *N* = 29	−1.1 (1.2)
Category fluency, z-score, *N* = 22	−1.6 (1.0)
Boston Naming Test, z-score, *N* = 19	−0.8 (1.0)
Rey-O Figure copy, z-score, *N* = 24	−1.8 (1.2)
Biomarkers	Median (IQR)
Neuron Specific Enolase (pg/ml), *N* = 35	24.4 (21.0–29.0)
Aβ1-42 (pg/ml), *N* = 51	353.7 (279.7–467.4)
Total Tau (pg/ml), *N* = 51	522.4 (368.1–998.6)
P-Tau_(181P)_ (pg/ml), *N* = 51	80.1 (60.2–104.7)
Amyloid Tau Index, *N* = 51	0.41 (0.25–0.58)
Amyloid PET SUVR, *N* = 22	2.41 (2.06–2.33)
Tau PET SUVR, *N* = 20	2.30 (1.86–2.77)
R Middle Frontal tau PET SUVR, *N* = 20	2.52 (1.64–3.25)
L Middle Frontal tau PET SUVR, *N* = 20	2.50 (1.78–3.29)
R Superior Parietal tau PET SUVR, *N* = 20	2.52 (1.74–2.94)
L Superior Parietal tau PET SUVR, *N* = 20	2.64 (2.06–2.92)
EEG	No. with positive finding/total tested (%)
Slowing; epileptiform abnormalities	14/17 (82%); 4/17 (24%)

a Five participants with homozygous allele, nine with heterozygous allele.

b A score of 20/38 on STMS is equivalent to an MoCA of 11/30 or MMSE of 19/30.

c Participants who could not complete Trails A (*n* = 6) were given a MOANS of 1 ∼ z-score of −3.

d Participants who could not complete Trails A did not have Trails B attempted and were not included in the z-score. Participants who could not complete Trails B in the maximum allotted time (*n* = 13) were given a MOANS of 1 ∼ z-score of −3.

AVLT DR = Rey Auditory Verbal Learning Test delayed recall; LOT = Learning Over Trials; RBD = REM Sleep Behaviour Disorder; Rey-O = Rey-Osterrieth Complex Figure; STMS = short test of mental status; WMS-III = Wechsler Memory Scale—3rd edition.

### Clinical and physical exam features

**Figure 1:**
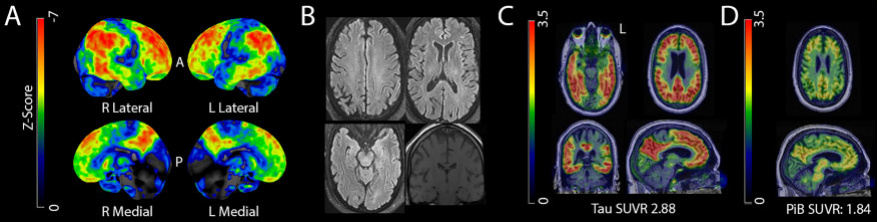
**Box 2 Example Case Neuroimaging** at presentation (2 years of symptoms) with (**A**) FDG–PET (Cortex ID, GE Healthcare) showing severe bilateral right > left temporal, parietal, precuneus, posterior cingulate and frontal hypometabolism; (**B**) MRI Axial T_2_ Flair and Coronal T_1_ images with mild right parietal atrophy with no frontal or hippocampal atrophy; (**C**) ^18^F-AV1451 tau PET with severe global tau radiotracer uptake; (**D**) Positive PiB amyloid-PET.

All 55 participants presented with cognitive predominant symptoms related to executive functions (example case in Box 2 and Fig. 1). Additionally, behavioural symptoms were present in 16/55 (29%) of participants but were not the predominant symptoms. Fifteen participants had apathy and one participant had impulsive or careless behaviours. Only two participants (3.6%) had mild Parkinsonism on exam, with one of these participants reporting REM sleep behaviour disorder (RBD). Overall, only two participants had RBD. Aphasia was described in 24/31 participants (77%) who exhibited features of logopenic aphasia with word-finding difficulties (circumlocution, lack of specificity and delays for word retrieval), phonological errors or difficulty with repetition. A speech pathology visit confirmed these findings in 6/6 participants. These participants did not meet root criteria for primary progressive aphasia due to language not being the predominant impaired domain or the presenting feature. Moderate to severe difficulties with Luria testing was described in 39/39 participants and 11/23 participants showed signs of simultanagnosia.

At presentation, nearly all participants were being evaluated for a second or third opinion regarding their diagnosis. In 16/55 participants, the outside working diagnosis was not listed or was not clearly defined (e.g. ‘cognitive impairment of unclear etiology’). In 7/55 participants the outside working diagnosis was fairly correct, labelling it ‘atypical or young onset Alzheimer’s disease’. Misdiagnosis was present in the other 32 participants: 14 diagnosed as a psychiatric (anxiety/depression) disorder, 8 diagnosed as frontotemporal dementia, 4 as autoimmune dementia (no antibodies detected) and failed intravenous steroid trials, 2 as primary progressive aphasia, 1 as corticobasal syndrome, 1 as arsenic poisoning (despite normal lab values), 1 as traumatic brain injury and 1 as chemotherapy-related dementia (lymphoma with no central nervous system involvement).

### Neuropsychological testing

Mean z-scores for each neuropsychological test are presented in Table 1. Some participants were administered an abbreviated battery to accommodate the severity of their cognitive deficits (sample sizes provided for each test in Table 1). Nineteen participants discontinued on Trails A (*n* = 6) or Trails B (*n* = 13) due to an inability to complete the test in the allowed time (180 and 300 s, respectively). To capture these deficits, we converted these participants’ discontinued scores to a MOANS scaled score of 1 (Machulda *et al.*, 2013), the equivalent of a z-score of −3. The six participants who discontinued on Trails A were not administered Trails B. Multi-domain cognitive impairment was evident, with the magnitude of impairment reflective of the task’s dependence on core executive functions (i.e. working memory, cognitive flexibility and inhibition). Participants had a mean DRS-2 of 107, which is associated with moderate difficulties in basic and instrumental activities of daily living (Fields *et al.*, 2010).

### Biomarker confirmation

**Figure 2:**
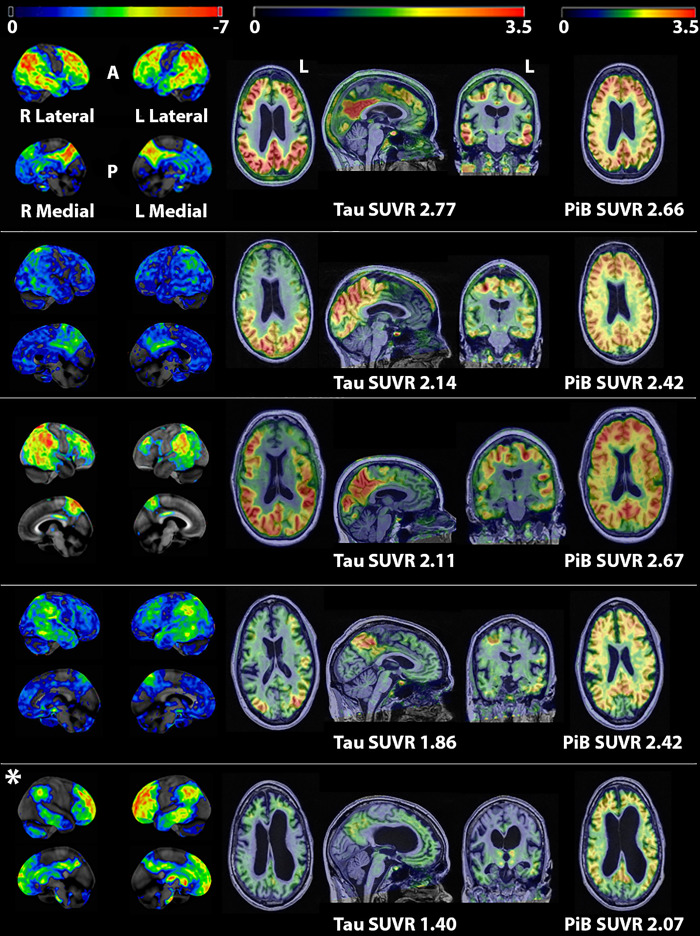
**Discordance of CSF p-tau biomarkers and tau PET** in five participants who had CSF p-tau in the normal range (< 61 pg/ml). *Left:* FDG–PET (using Cortex ID, GE Healthcare) with z-score bar at the top. *Middle:*^18^F-AV1451 tau PET with SUVR bar at the top and individual meta-ROI SUVR below each image. *Right:* PiB amyloid PET with SUVR bar at the top and individual meta-ROI SUVR below each image. Significant ^18^F-AV1451 uptake was present in all five participants (mean SUVR 2.05). CSF p-tau level, tau PET SUVR and mean time lag between CSF and tau PET for each participant can be found in Supplementary Table 1. *Bottom participant came to autopsy and was found to have A3, B3, C3 Alzheimer’s disease pathology.

Biomarker confirmation was established via CSF biomarkers alone (32 participants), CSF biomarkers and positive amyloid and tau PET (15 participants), positive amyloid and tau PET (3 participants), CSF biomarkers and amyloid-PET (2 participants), amyloid-PET (1 participant), autopsy with all biomarker modalities available (1 participant) or autopsy only (1 participant). All participants with CSF testing had an Amyloid Tau Index <1 (median 0.41, IQR 0.25–0.58). Twelve (24%) participants had CSF p-tau below the cut-off value of <61 pg/ml specific to this assay. Five of these participants had tau PET scans, all of which were unequivocally positive with a mean SUVR of 2.05 and are highlighted in Fig. 2. One additional participant with low CSF p-tau and positive tau PET came to autopsy and had pathology proven Alzheimer’s disease.

### Electroencephalogram

Seventeen participants underwent a routine 30-min EEG. In 10 participants, the indication for EEG was to work-up early-onset dementia, whereas the 7 remaining participants had atypical episodic symptoms that prompted EEG testing. Of the 14/17 participants with abnormal EEGs, 13 had diffuse slowing (2 participants with superimposed frontal rhythmic delta activity). Three participants had epileptiform discharges arising from the central midline (one participant) and bi-temporal (two participants) regions.

### Neuroimaging

**Figure 3:**
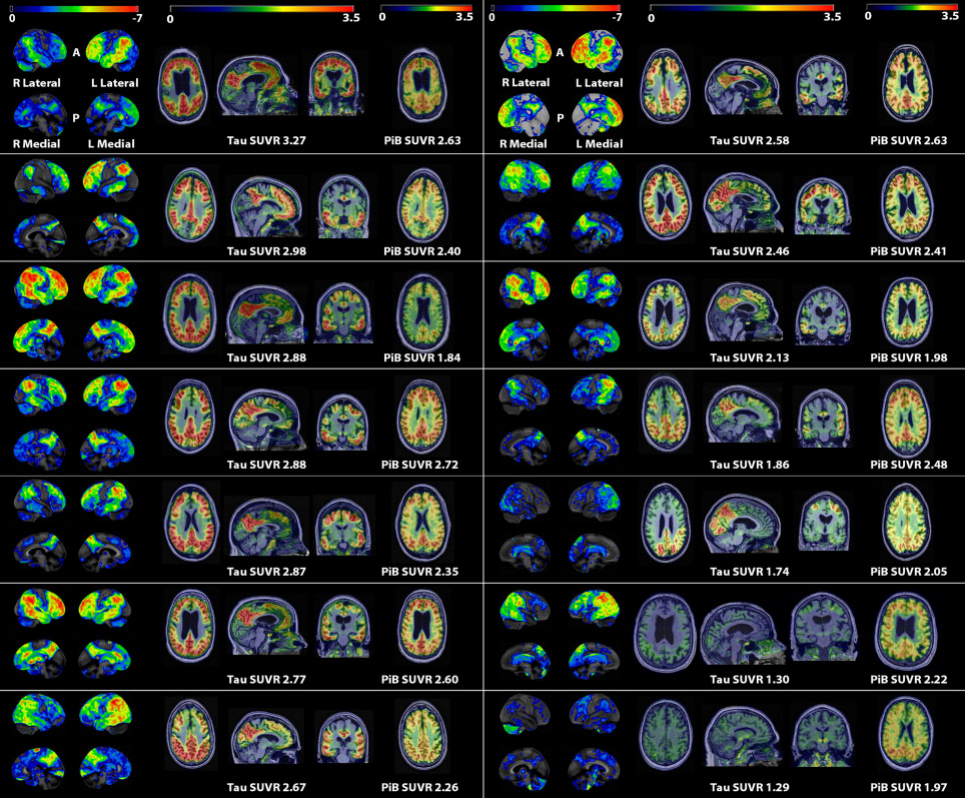
**Molecular neuroimaging and heterogeneity in dysexecutive Alzheimer’s disease **of the remaining participants with all three molecular imaging modalities available (participant in Fig. 5 not included). Participants are separated into two columns. Within each column: *Left:* FDG–PET (using Cortex ID, GE Healthcare) with z-score bar at the top. *Middle:*^18^F-AV1451 tau PET with SUVR bar at the top and individual meta-ROI SUVR below each image. *Right:* PiB amyloid-PET with SUVR bar at the top and individual meta-ROI SUVR below each image. There is hypometabolism and tau PET uptake predominantly in the dorsolateral prefrontal, lateral temporoparietal, precuneus and posterior cingulate regions, with inter-individual heterogeneity in topography and degree of involvement. CSF p-tau level, tau PET SUVR and mean time lag between CSF and tau PET for each participant can be found in Supplementary Table 1.

All 55 participants underwent MRI and FDG–PET, 22 participants underwent amyloid-PET and 20 participants underwent tau PET (in addition to amyloid PET). Frontal atrophy was minimal on visual inspection, but FDG–PET frontal hypometabolism was prominent in many cases. Lateral parietal, dorsolateral prefrontal, precuneus and posterior cingulate regions were most commonly involved, which visually overlap with regions of ^18^F-AV-1451 uptake (Figs 2 and 3). To best represent the heterogeneity in the patterns observed, we present neuroimaging findings on the single-subject level in Fig. 3.

**Figure 4:**
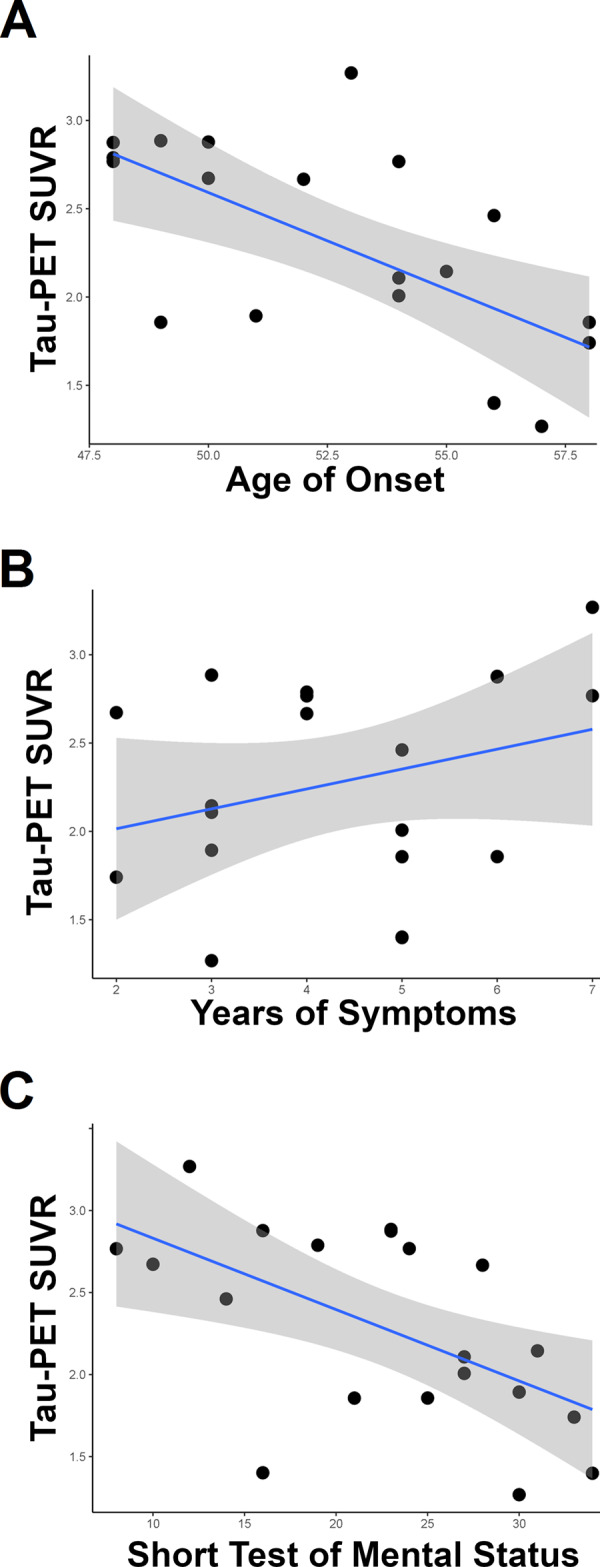
**Age of onset association with tau PET burden:** Scatter plots of the 20 participants with tau PET scans: (**A**) A younger age of onset was associated with a higher tau PET SUVR (multiple *r*^2^ = 0.41; adjusted *r*^2^ = 0.38, *P* < 0.005). (**B**) Years of symptoms prior to tau PET was not statistically significant (multiple *r*^2^ = 0.09; adjusted *r*^2^ = 0.04, *P* = 0.21). (**C**) Higher tau PET SUVR was associated with lower bedside cognitive testing (multiple *r*^2^ = 0.33; adjusted *r*^2^ = 0.29, *P* < 0.01).

Amyloid PET was positive for all 22 participants who had imaging and the median SUVR was 2.41 (IQR: 2.06–2.33). The topography followed previously documented Alzheimer’s disease patterns across all participants (Fripp *et al.*, 2008). The 20 participants who underwent Tau PET were all positive and had a median SUVR was 2.30 (IQR: 1.86–2.77). Exploratory analysis reveals that an earlier age of onset was associated with higher global tau PET SUVRs (multiple *r*^2^ = 0.41; adjusted *r*^2^ = 0.38, *P* < .005) and remained significant when controlling for years of symptoms prior to tau PET scan (multiple *r*^2^ = 0.39, adjusted *r*^2^ = 0.36, *P* < .01) (Fig. 4A and B). The middle frontal and superior parietal lobes demonstrated the highest tau PET SUVR loads and were fairly symmetric at the group level (Table 1). Increased global tau PET SUVR was associated with increased cognitive impairment on bedside exam (multiple *r*^2^ = 0.33; adjusted *r*^2^ = 0.29, *P* < .01) (Fig. 4C). Increased global amyloid PET SUVR was not associated with increased cognitive impairment on bedside exam (multiple *r*^2^ = 0.11; adjusted *r*^2^ = 0.06, *P* = .15).

**Figure 5:**
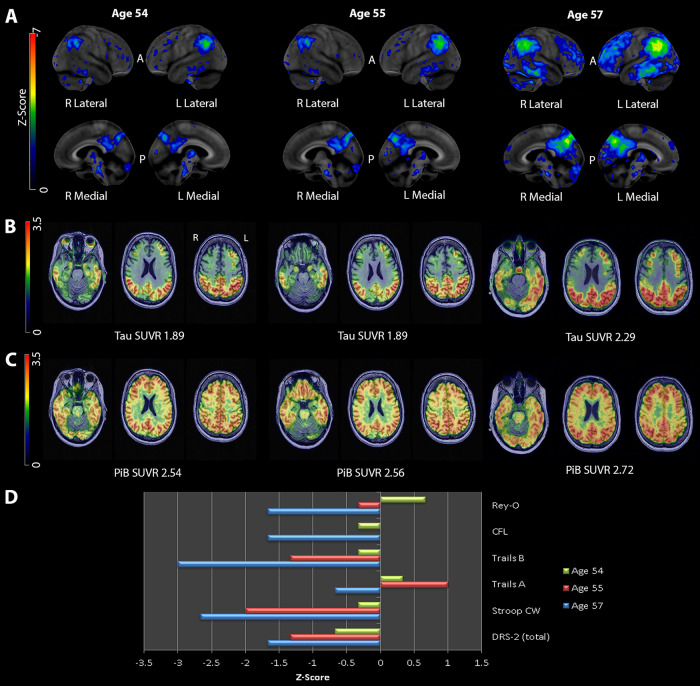
**Longitudinal case example:** A 54-year-old female with 3 years of mild multi-tasking difficulties before presentation had 3 years of longitudinal molecular imaging with (**A**) FDG–PET (Cortex ID z-score 0 to −7, GE Healthcare) showing mild hypometabolism that progresses over 3 years. (**B**) ^18^F-AV1451 tau PET (SUVR scale 0–2.5) radioligand uptake preceding hypometabolism at each time point. (**C**) Significant PiB amyloid-PET (SUVR scale 0–3) radioligand uptake in an Alzheimer’s disease pattern. (**D**) Neuropsychological scores showing mild impairment at initial presentation (green bars) despite already significant tau PET uptake in the bilateral temporal, parietal and frontal lobes. Cognitive impairment progressed at two-time points (red and blue bars) consistent with FDG–PET and tau PET progression. Trails B and Stroop Color-Word testing were severely impaired and multiple cognitive domains were impaired after 3 years. Logical memory based tests were also significantly abnormal but orientation questions on MoCA were not altered until the third time point (6/6, 6/6, 4/6 at each visit). DRS-2, Dementia Rating Scale-2; Rey-O; SUVR, standardized uptake value ratio.

In one participant with longitudinal scans, the severity of ^18^F-AV-1451 uptake preceded a similar pattern of FDG hypometabolism (Fig. 5).

### Neuropathology

**Figure 6:**
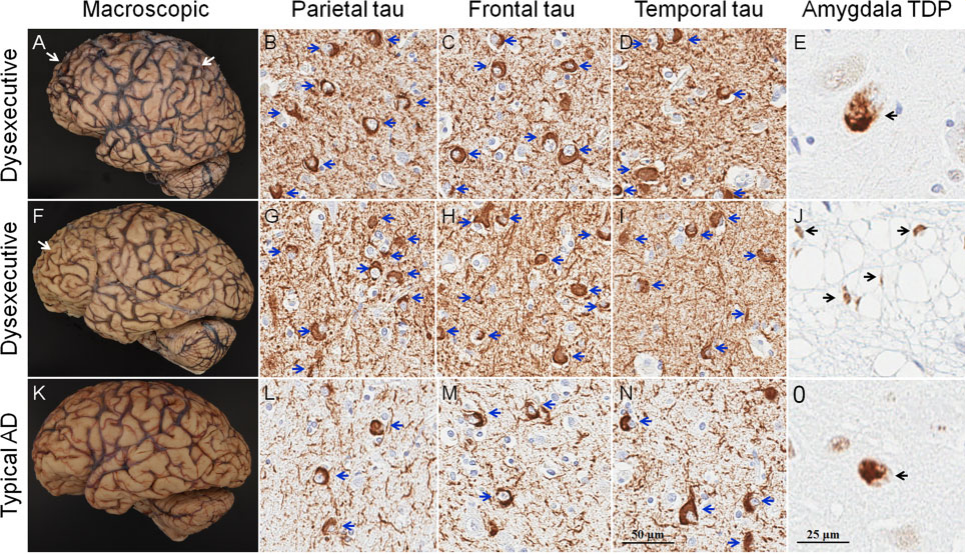
**Autopsy results from two dysexecutive Alzheimer’s disease participants and one typical Alzheimer’s disease participant.** (**A–E**) A 54-year-old female had 7 years of progressive symptoms that started with organizing/planning difficulties which lead to losing her job. She additionally repeated stories and had difficulty with numbers. Her initial MRI 2 years into symptoms showed moderate left parietal, mild right parietal and left frontal atrophy. Within 6 years her cognition rapidly decreased and her MoCA was zero. On autopsy, she had a Thal amyloid phase of 5 and Braak tangle stage of VI. (**A**) Upon macroscopic inspection, significant global atrophy was observed affecting parietal and frontal lobes to a greater extent compared to the temporal lobe. (**B**) Tau pathology was greatest in parietal and (**C**) frontal cortex compared to (**D**) temporal cortex. (**E**) TDP-43 immunohistochemistry in the amygdala revealed sparse neuronal cytoplasmic inclusions and neurites. (**F–J**) A 62-year-old male participant had 7 years of progressive symptoms that started with impairment in multi-tasking and problem-solving. Over time he developed severe aphasia and apraxia on the right side. (**F**) Macroscopic inspection revealed global atrophy, predominantly involving the frontal lobe. He had a Thal amyloid phase of 5 and Braak tangle stage of V. (**G**) The amount of tau pathology observed in the parietal and (**H**) frontal cortices was greater than that observed in (**I**) temporal cortex. (**J**) TDP-43 immunohistochemistry in the amygdala revealed subpial neurites, but no neuronal cytoplasmic inclusions were observed. (**K–O**) An 84-year-old male was first noted to have symptoms of memory loss with a slow, but significant progression to typical Alzheimer’s dementia. (**K**) Macroscopic inspection revealed global atrophy without focal lobar involvement. (**L**) Tau pathology was observed in parietal and (**M**) frontal cortices to a lesser extent than that observed in (**N**) temporal cortex. (**O**) TDP-43 immunohistochemistry in the amygdala revealed sparse neuronal cytoplasmic inclusions and neurites. Scale bar represents 50 μm for tau photomicrographs and 25 μm for TDP-43 photomicrographs.

Both participants who came to autopsy had a high level of Alzheimer’s disease neuropathologic change per the National Institute on Aging-Alzheimer’s Association 2012 consensus guidelines. The hippocampus was relatively spared compared to frontal and parietal cortex (Fig. 6). The male decedent had evidence of aging-related tau astrogliopathy with clusters of thorn-shaped astrocytes observed in the white matter. Neither participant had evidence of alpha-synuclein-immunoreactive lesions or significant cerebrovascular disease. Microscopic inspection of the amygdala revealed TDP-43 positive neuronal cytoplasmic inclusions in one of the participants and subpial TDP-43 positive neurites in the other participant.

### Group-wise comparison across Alzheimer’s disease phenotypes enrolled in the Mayo ADRC

**Figure 7:**
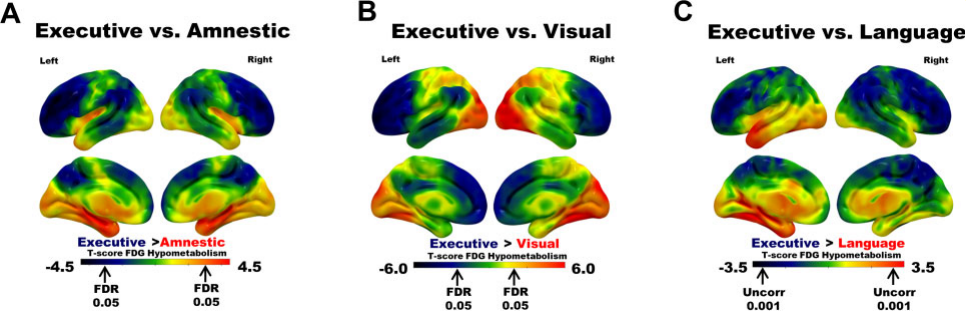
**FDG–PET in executive predominant presentations versus amnestic, visual and language predominant Alzheimer’s disease phenotypes.** The FDG–PET scans from participants with dysexecutive predominate presentations (*n* = 15) were compared to (**A**) amnestic predominate (*n* = 110), (**B**) visual predominant (*n* = 18), (**C**) and language predominant (*n* = 7) presentations of Alzheimer’s disease. In each panel, the blue-black end of the spectrum encodes the *t*-score for a greater degree of hypometabolism in the dysexecutive phenotype with red-orange encoding the greater degree of either (**A**) amnestic, (**B**) visual or (**C**) language phenotype. The *t*-value corresponding to voxel-level *P*-value of FDR corrected 0.05 (**A** and **B**) or uncorrected 0.001 (**C**) is indicated with arrows in the colour-bar.

Comparing FDG–PET in participants with the executive predominate presentation of Alzheimer’s disease relative to other Alzheimer’s disease phenotypes revealed unique areas of relatively greater hypometabolism in parieto-frontal cortex and relative sparing of the medial temporal lobe (versus amnestic phenotype), occipital (versus visual phenotype) and left temporal (versus language phenotype) (Fig. 7).

**Figure 8:**
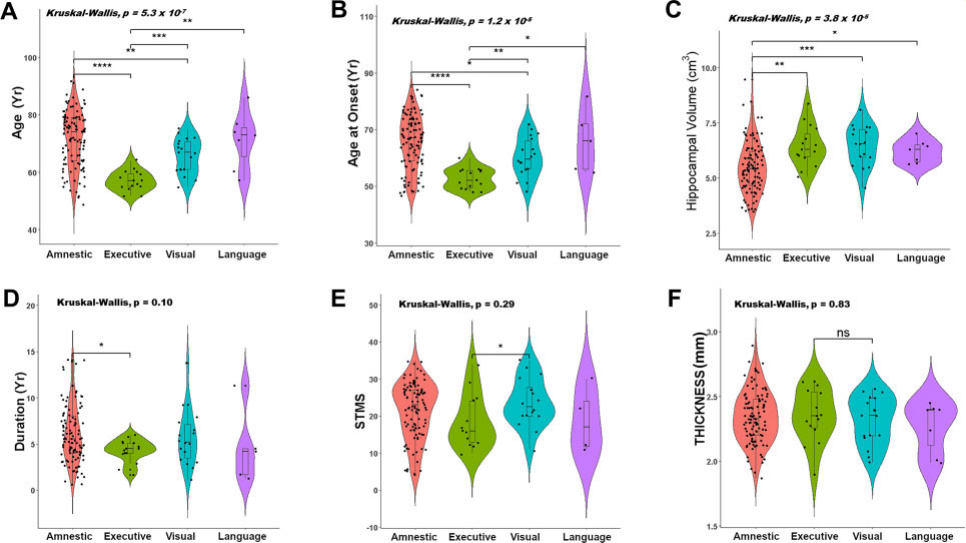
**Violin scatter plots comparing variables** between executive (*n* = 15), amnestic (*n* = 110), visual (*n* = 18) and language predominant (*n* = 7) Alzheimer’s disease phenotypes. There was a significant main effect of phenotype for (**A**) age at evaluation, (**B**) age at disease onset and (**C**) hippocampal volume. There was no significant main effect of phenotype for (**D**) disease duration, (**E**) short test of mental status (STMS), (**F**) or cortical thickness in Alzheimer’s disease signature regions. Pairwise *P*-values are indicated as follows: ns = 1, *0.05, **0.01, ***0.001, ****0.

A comparison between groups revealed significant differences in age, age at onset, and hippocampal volume (Fig. 8A–C). There was no difference in disease duration, bedside cognition or cortical thickness in Alzheimer’s disease signature regions (Fig. 8D–G). Given the differences in age by phenotype, we repeated the voxel-wise analysis of the FDG–PET images while controlling for age. This altered the statistical thresholds but the anatomic patterns of the relative difference between groups remained the same (data not shown).

## Discussion

We describe a large group of participants with a progressive dysexecutive syndrome that is attributable to Alzheimer’s disease. In our cohort, nearly all participants were affected during their productive working years at symptom onset, and it took an average of 3 years before they received an accurate diagnosis by a behavioural neurologist. Initial misdiagnosis was common. When clear documentation was available 32 of 39 cases were initially misdiagnosed. Diagnostic confusion can arise from the younger age of onset, the neuropsychological profile, and neuroimaging and biomarker findings. As our example case (Box 2) illustrates, there can exist discordance between a patient’s previous high levels of function with the presence of severe multi-domain impairment on cognitive testing. On average, bedside testing in our cohort was in the moderate to severe dementia range with a mean STMS of 20/38, which is equivalent to a Montreal Cognitive Assessment score of 11/30 (Townley *et al.*, 2019) and an MMSE score of 19/30 (Tang-Wai *et al.*, 2003). Compared to the degree of clinical impairment, MRI atrophy can be subtle at presentation and most apparent in parietal regions rather than the medial temporal lobe.

This pattern of atrophy has been reported previously (Dickerson *et al.*, 2011; Mendez *et al.*, 2012; Scheltens *et al.*, 2017) but diagnosis can be obscured if one is looking for a typical Alzheimer’s disease pattern. Even when a neurodegenerative aetiology is suspected, ancillary tests can create additional diagnostic uncertainty. CSF testing demonstrated normal p-tau in a subset of participants (24% in our cohort), and FDG–PET commonly demonstrated significant frontal hypometabolism (Fig. 7), which can be misidentified as frontotemporal dementia. Based on our provisional diagnostic criteria (Box 1), a diagnosis of dysexecutive Alzheimer’s disease represents a progressive dysexecutive syndrome with a positive biomarker profile along the Alzheimer’s continuum (Jack *et al.*, 2018). According to the NIA-AA research criteria framework, our seven participants with abnormal amyloid (A+) but normal CSF p-tau (T−) and no tau PET available are most accurately described as *consistent* with Alzheimer’s neuropathologic change rather than *definitive* progressive dysexecutive syndrome with Alzheimer’s disease. Once tau PET becomes clinically available, it could help address any diagnostic uncertainty in patients with normal CSF p-tau levels (Fig. 2).

Similar to prior studies, atrophy on MRI was mild to moderate in lateral parietal cortices but only mild in frontal cortices (Dickerson *et al.*, 2011; Ossenkoppele *et al.*, 2015). Despite this, FDG–PET and tau PET imaging demonstrated significant hypometabolism and tau radiotracer uptake in the bilateral frontal and lateral parietal cortices. We note that one participant with 3 years of longitudinal data available showed FDG–PET hypometabolism lagging tau PET abnormalities (Fig. 5). Although autopsies are cross-sectional in nature, intra-individual comparisons may provide some insight. Neuropathologic examination revealed significant tau pathology in parieto-frontal cortices with relative hippocampal sparing (Murray *et al.*, 2011) in a pattern mirroring the FDG–PET abnormalities (Fig. 7) and differences in hippocampal volume (Fig. 8C). The interplay and sequencing of amyloid, tau, metabolism, atrophy and clinical symptoms in participants with dysexecutive Alzheimer’s disease are uncertain but are areas of ongoing investigation.

Prior studies have reported conflicting results regarding the involvement of frontal versus parietal regions on molecular imaging in dysexecutive Alzheimer’s disease. One study examined FDG–PET and tau PET in two participants diagnosed with dysexecutive Alzheimer’s disease and showed frontal, parietal and temporal lobe hypometabolism that topographically overlapped with ^18^F-AV-1451 uptake (Dronse *et al.*, 2017), whereas another study described one participant with dysexecutive/behavioural mixed phenotype with parietal ^18^F-AV-1451 uptake and no frontal uptake (Ossenkoppele *et al.*, 2016). In another, participants with various Alzheimer’s disease phenotypes were described and executive impairment correlated with dorsolateral frontal and lateral parietal tau PET signal (Bejanin *et al.*, 2017). From a molecular imaging standpoint, our study builds upon and reconciles these discordant studies in three ways: (i) although the most common metabolic and tau PET pattern involved both frontal and parietal regions, participants with parietal-predominant involvement also presented with a dysexecutive syndrome, (ii) executive impairment, whether associated with dysexecutive Alzheimer’s disease or other Alzheimer’s disease phenotypes, is associated with lateral prefrontal and parietal tau PET uptake and (iii) like all forms of Alzheimer’s disease, there is significant inter-individual heterogeneity in the pattern of molecular imaging that is associated with a dysexecutive Alzheimer’s disease phenotype (Fig. 3). Future studies should emphasize phenotypic subtypes that may meet the criteria for dysexecutive Alzheimer’s disease.

The factors that drive syndromic diversity and heterogeneity within a specific atypical Alzheimer’s disease syndrome are not well understood. According to the network model of Alzheimer’s disease, genetic and environmental factors may influence regional cortical atrophy patterns (Wolk *et al.*, 2010; Scheltens *et al.*, 2017) and may predispose a selective network to vulnerability (Warren *et al.*, 2012). The pattern of network failure in an individual then drives the clinical and anatomic syndromic diversity (Jones *et al.*, 2017). In our early-onset cohort, the dysexecutive phenotype appeared to be associated with disruption of bilateral parieto-frontal regions, which is consistent with prior early-onset Alzheimer’s disease studies describing executive impairment (Dickerson *et al.*, 2011, 2017; Daianu *et al.*, 2016; Scheltens *et al.*, 2017). We recently described a parieto-frontal tau-PET pattern using a data-driven method and found a similar association with younger age of Alzheimer’s disease dementia onset and tau deposition in this system that spatially overlaps with the working memory network (Jones *et al.*, 2017).

Definitions of executive functions vary, but there is general agreement that there are three core dissociable abilities: (i) working memory (monitoring existing and updating incoming information), (ii) cognitive flexibility (incorporating simultaneous streams of information and set-shifting between mental tasks) and (iii) cognitive inhibitory control (suppressing irrelevant incoming information) (Miyake *et al.*, 2000; Baddeley, 2012; Diamond, 2013). Of note, though simple temporary storage of information is a component of working memory (often referred to as short-term memory), it is the utilization and manipulation of this information for completion of tasks/goals that are characteristic of working memory as it relates to executive function (Baddeley, 2012).

Many participants used ‘memory trouble’ to describe their cognitive difficulties but with further questioning often described difficulties with multi-tasking, completing tasks with multiple steps, playing board games with family, following directions/recipes, learning new computer software, mental calculations, organizing personal calendars, or planning and executing projects at home or work, suggesting more executive dysfunction with prominent deficits in working memory function.

The parieto-frontal regions form an executive network (Selemon and Goldman-Rakic, 1988) that helps coordinate attentional selectivity and heavily influences working memory capacity (Rottschy *et al.*, 2012). These networks also have a high degree of between-subject variability (Marek and Dosenbach, 2018). Impairment in executive control tests, like the Stroop Color-Word test, can be predicted by individual variation in working memory capacity and individuals with low working memory capacity had difficulty with the Stroop interference trial by two separate but complementary mechanisms (Kane and Engle, 2003). Attentional processes needed for competition resolution can only be engaged when working memory has sufficiently maintained the task/goal (i.e. ‘ignore the word and respond to color’).

The working memory network can be separated into specialized verbal working memory (phonologic loop) and visual-spatial working memory (visuospatial sketchpad) that further interact with a central executive system, which controls and regulates cognitive processes to promote the primary executive functions described above (Baddeley, 1992; Baddeley, 2012). There is conflicting evidence on lateralized contributions to these subsystems, but most evidence suggests there is bilateral activation of parieto-frontal networks in working memory tasks with specialized contributions from the left and right hemisphere depending on the task involved (Gur *et al.*, 1994; Cabeza and Nyberg, 2000; Walter *et al.*, 2003; Ray *et al.*, 2008). In a study evaluating visual working memory, the bilateral intraparietal sulci were central hubs and the strength of network synchrony in this region predicted visual working memory load capacity (Palva *et al.*, 2010). It is reasonable to hypothesize that severe dysfunction of central hubs in large-scale networks may explain the diffuse slowing seen on 14/17 EEGs in our cohort. Future studies combining electrophysiological monitoring with functional neuroimaging would help test this hypothesis.

Features of logopenic aphasia were common in our cohort (24/31) and the phonological loop (Baddeley, 1996), responsible for the rehearsal of auditory input, overlaps with the left working memory network (Jones *et al.*, 2012). Impaired functional connectivity in the left working memory network has been associated with impaired repetition in patients with logopenic aphasia (Whitwell *et al.*, 2015). Tau deposition also occurs in the left working memory network to a greater degree in patients with younger onset Alzheimer’s disease (Jones *et al.*, 2017). Despite having features of logopenic aphasia with FDG–PET and tau-PET findings consistent with abnormalities in the left working memory network brain regions, the participants included in our study did not meet root criteria for primary progressive aphasia because language was not the predominant deficit and was not the initial symptom described (Gorno-Tempini *et al.*, 2011). Our patients also displayed wide-spread cognitive dysfunction in multiple domains without a particular predilection for confrontation naming (Table 1).

Tests of auditory verbal memory—including WMS-R/III Logical memory and the AVLT—are not traditionally considered executive functioning tasks. However, working memory, particularly the phonological loop, is required for holding onto that information in the short term and organizing content for learning (i.e. encoding) and later recall. Thus, deficits in working memory would feasibly produce impairments in the ability to encode the information. Indeed, a prominent difficulty of encoding on the AVLT has been described in early-onset Alzheimer’s disease (Dickerson *et al.*, 2017) and is consistent with working memory network impairment involving the phonologic loop (Buchsbaum *et al.*, 2011; Stopford *et al.*, 2012). Similar to our findings, Dickerson *et al.* reported relative preservation of semantic memory (i.e. confrontation naming), supporting a theoretical framework that phonologic and semantic-based networks are separable to some degree (Baddeley, 2012). Importantly, although memory retention was impaired across all measures (i.e. AVLT DR, LM II, VR II), it was not the defining feature of the presentation.

Despite evident executive dysfunction, participants in our cohort had surprisingly relatively less impairment on one measure of working memory: the combined forward and backward digit span (z-score = −1.0). Forward digit span is a simple auditory attention task (i.e. not working memory) while backward digit span is the working memory portion of the task (Kaiser *et al.*, 2012; Egeland, 2015). We would not expect as much impairment on the forward digit span task as it measures only simple attention but backwards digit span could not be separated from the combined score using our normative data (norms not available for WMS-III or R). Therefore, difficulties in this subtest may be underrepresented in the combined score. In line with this hypothesis, Letter Number Sequencing, another working memory test, was notably worse (z-score = −1.7).

Similar to previous studies (Scheltens *et al.*, 2017), disproportionate impairment was evident on Trails B and 13/22 participants were unable to complete the task in the allotted 300 s (an additional 6 participants did not attempt Trails B due to failure to complete Trails A), creating a floor effect. This test requires not only visual processing but also mental set shifting throughout the test. It could be argued that difficulties on Trails B may involve some disruption of the dorsal visual stream (Kravitz *et al.*, 2011). However, participants with dysexecutive Alzheimer’s disease had normal simultanagnosia testing (Thomas *et al.*, 2012) in over 50% of documented cases, which is a sensitive test for dorsal stream dysfunction. Additionally, participants maintained a better ability to complete Trails A (22/28 completed), which does not require task set-shifting (i.e. performance on Trails A versus Trails B in Fig. 5). All 39 participants tested with Luria motor sequence had moderate to severe difficulties executing this sequential task, including the 12 participants who tested normally on simultanagnosia.

Separate from Luria testing, praxis errors with ideomotor apraxia were also documented in 28 out of 40 cases. The large-scale parieto-frontal-basal ganglia network model of apraxia (Gross and Grossman, 2008) would be consistent with dysfunction in the working memory network needed to conceptualize, plan and execute motor tasks in these participants (Króliczak *et al.*, 2016; Matt *et al.*, 2017). The inability to perform tasks under executive control was the main limiting factor impacting activities of daily living in this cohort, and counselling to avoid multi-tasking, environmental and emotional distractions, and emphasize strategies to facilitate sequential processing to improve daily task performance appeared to be anecdotally beneficial. While the current findings offer an important clue to the difficulties these participants experience in sequential processing, further research with standardized assessments and formal criteria for simultanagnosia, praxis and Luria testing are needed to better understand the frequency and predictive values of abnormalities for these tests in dysexecutive Alzheimer’s disease and the implications they may have on management and counselling.

Overall, the multi-domain nature of the cognitive dysfunction observed in this cohort is consistent with impaired executive functioning, particularly working memory. This type of impairment would be expected to impact cognitive performance on a wide range of mentally effortful tasks that require conscious active manipulation of abstract and/or simultaneous information streams. These deficits in working memory and cognitive flexibility have long been observed in Alzheimer’s disease cohorts in cross-sectional (Baddeley, 1986) and longitudinal studies (Baddeley *et al.*, 1991), but in the participants described here, executive dysfunction is the core defining clinical feature. This dysfunction is related to specific disruption of brain regions involved in executive functions (Baddeley, 2003) with relative sparing of brain regions supporting other functions (e.g. memory, vision and language) (Fig. 7). These features cannot be explained by differences in disease duration or severity (Fig. 8).

Broad behavioural and personality changes were not a feature of this cohort. Apathy was the most common behavioural symptom documented at presentation (29%) and is likely related to overall executive dysfunction (Boyle *et al.*, 2003; Barnes *et al.*, 2015; Lohner *et al.*, 2017). Disinhibited behavioural symptoms and inhibition problems, in general, were not a defining feature of this cohort, in contrast to abnormalities in other core executive functions (e.g. working memory and cognitive flexibility). This lack of significant dis-inhibition and personality changes may prove to be a differentiating factor between dysexecutive Alzheimer’s disease, behavioural variant frontotemporal dementia and perhaps even behavioural Alzheimer’s disease. Further studies directly comparing these syndromes will be needed to assess the predictive value of behavioural changes as they relate to the primary brain networks involved.

CSF studies demonstrated that 24% of participants had p-tau below the nominal threshold (<61 pg/ml). This threshold is based on enzyme-linked immunosorbent assay testing in clinically diagnosed Alzheimer’s disease from non-Alzheimer’s disease causes of mild cognitive impairment or dementia (Humpel, 2011; Simonsen *et al.*, 2017). Although it is possible that some of our participants with abnormal amyloid biomarkers and low CSF p-tau levels had an additional non-Alzheimer’s disease cause of their cognitive impairment, 5 of these 12 participants underwent tau PET, all of which were unequivocally positive (mean SUVR 2.05). One of these participants also underwent an autopsy and had Alzheimer’s disease neuropathological change (Fig. 6F–J). Decreasing levels of CSF p-tau prior to and after symptom onset has been recently described in Dominantly Inherited Alzheimer’s Network (DIAN) participants (McDade *et al.*, 2018). Amyloid plaque development is theorized to sequester detectable CSF amyloid, and through a similar mechanism, neurofibrillary tangle accumulation has been proposed to result in lower CSF p-tau values at symptom onset (McDade *et al.*, 2018). Potentially supporting this hypothesis, participants with dysexecutive Alzheimer’s disease often presented with significantly elevated tau PET uptake suggestive of significant neurofibrillary tangle burden (Smith *et al.*, 2016). We did not find any direct correlation with tau PET SUVR and CSF p-tau level, but this is likely complicated by lag time between the diagnostic measures (median time to tau PET after CSF was 12 months, IQR 5–20 months) and limited statistical power (*n* = 17). Regardless of the underlying reason, clinicians should not discount the possibility of Alzheimer’s disease as an aetiology based on the absence of elevated CSF p-tau. Future studies re-examining the accuracy of these cut-off values with regards to disease stage, the age of onset, confirmation via *in vivo* molecular imaging and post-mortem examination would further enhance our understanding in this regard.

Limitations of the present study include its lack of standardized clinical assessments. For example, although we found that apraxia and aphasia were highly prevalent, these were not universally documented, raising the possibility that negative or subtle symptoms went undocumented. Similarly, several participants did not undergo neuropsychological testing, EEG, genetic testing or tau PET imaging, bringing up the possibility of selection bias and a non-representative sample for participants who had these tests performed. The majority of these participants were being evaluated for second or third opinions, which may represent a selection bias that could potentially skew the prevalence of misdiagnosis and the biomarker profiles described in this report. The retrospective nature of this study also makes it difficult to know how the results impacted clinical interpretations. Although, the strikingly consistent anatomic pattern of neurodegeneration and tau pathology in parieto-frontal brain regions key for performing tasks under executive control provide *post hoc* validity to the clinical construct of a progressive dysexecutive syndrome as defined here. Future prospective studies will be needed for a higher level of validation. Another limitation of this study is the lack of a comparison group recruited in the same way in which to estimate the sensitivity and specificity of our proposed criteria. However, this represents an initial effort in formulating diagnostic criteria for a progressive dysexecutive syndrome and further classification for those with an aetiology attributable to Alzheimer’s disease. Future studies with comparison groups will be necessary before consensus criteria can be derived.

Progressive dysexecutive syndrome due to Alzheimer’s disease was characterized by early, predominant and progressive cognitive dysfunction in executive tasks with less prominent behavioural changes. Whereas patients can be significantly impaired on cognitive tests, MRI demonstrates only subtle changes early on and FDG–PET is important to consider in accurately diagnosing these patients. Parietal lobe involvement is more characteristic than medial temporal lobe involvement which may appear unaffected in some patients. Falsely negative CSF p-tau levels were not uncommon, and future studies involving tau PET will be important to confirm more cases of false-negative CSF values.

## Supplementary Material

fcaa068_Supplementary_Data

## Abbreviations

ARDC =: Alzheimer’s Disease Research Center
FDG =: ^18^F-fluorodeoxyglucose
MMSE =: Mini-Mental Status Exam
MOANS =: Mayo Older Americans Normative Studies
MoCA =: Montreal Cognitive Assessment
NIA-AA =: National Institute on Aging—Alzheimer’s Association
ROI =: region of interest
STMS =: short test of mental status; SUVR = standard uptake value ratio

## References

[fcaa068-B1] Andreasen N, Minthon L, Davidsson P, Vanmechelen E, Vanderstichele H, Winblad B, et al Evaluation of CSF-tau and CSF-Abeta42 as diagnostic markers for Alzheimer disease in clinical practice. Arch Neurol 2001; 58: 373–9. 11255440 10.1001/archneur.58.3.373

[fcaa068-B2] Ashburner J, Friston KJ. Unified segmentation. Neuroimage 2005; 26: 839–51. 15955494 10.1016/j.neuroimage.2005.02.018

[fcaa068-B3] Baddeley A. Working memory Vol. 11 Oxford: Oxford University Press, Clarendon Press; 1986.

[fcaa068-B4] Baddeley A. Working memory. Science 1992; 255: 556–9. 1736359 10.1126/science.1736359

[fcaa068-B5] Baddeley A. The fractionation of working memory. Proc Natl Acad Sci U S A 1996; 93: 13468–72. 8942958 10.1073/pnas.93.24.13468 PMC33632

[fcaa068-B6] Baddeley A. Working memory: looking back and looking forward. Nat Rev Neurosci 2003; 4: 829–39. 14523382 10.1038/nrn1201

[fcaa068-B7] Baddeley A. Working memory: theories, models, and controversies. Annu Rev Psychol 2012; 63: 1–29. 21961947 10.1146/annurev-psych-120710-100422

[fcaa068-B8] Baddeley AD, Bressi S, DELLA Sala S, Logie R, Spinnler H. The decline of working memory in Alzheimer’s disease: a longitudinal study. Brain 1991; 114: 2521–42. 1782529 10.1093/brain/114.6.2521

[fcaa068-B9] Barnes J, Dickerson BC, Frost C, Jiskoot LC, Wolk D, van der Flier WM. Alzheimer’s disease first symptoms are age dependent: evidence from the NACC dataset. Alzheimers Dement 2015; 11: 1349–57. 25916562 10.1016/j.jalz.2014.12.007 PMC4619185

[fcaa068-B10] Bejanin A, Schonhaut DR, La Joie R, Kramer JH, Baker SL, Sosa N, et al Tau pathology and neurodegeneration contribute to cognitive impairment in Alzheimer’s disease. Brain 2017; 140: 3286–300. 29053874 10.1093/brain/awx243 PMC5841139

[fcaa068-B11] Boyle PA, Malloy PF, Salloway S, Cahn-Weiner DA, Cohen R, Cummings JL. Executive dysfunction and apathy predict functional impairment in Alzheimer disease. Am J Geriatr Psychiatry 2003; 11: 214–21. 12611751

[fcaa068-B12] Braak H, Braak E. Neuropathological stageing of Alzheimer-related changes. Acta Neuropathol 1991; 82: 239–59. 1759558 10.1007/BF00308809

[fcaa068-B13] Brazis PW, Graff-Radford NR, Newman NJ, Lee AG. Ishihara color plates as a test for simultanagnosia. Am J Ophthalmol 1998; 126: 850–1. 9860021 10.1016/s0002-9394(98)00187-1

[fcaa068-B14] Buchsbaum BR, Baldo J, Okada K, Berman KF, Dronkers N, D’esposito M, et al Conduction aphasia, sensory-motor integration, and phonological short-term memory–an aggregate analysis of lesion and fMRI data. Brain Lang 2011; 119: 119–28. 21256582 10.1016/j.bandl.2010.12.001 PMC3090694

[fcaa068-B15] Cabeza R, Nyberg L. Imaging cognition II: an empirical review of 275 PET and fMRI studies. J Cogn Neurosci 2000; 12: 1–47. 10.1162/08989290051137585 10769304

[fcaa068-B16] Crutch SJ, Schott JM, Rabinovici GD, Murray M, Snowden JS, van der Flier WM, et al; Alzheimer's Association ISTAART Atypical Alzheimer's Disease and Associated Syndromes Professional Interest Area. Consensus classification of posterior cortical atrophy. Alzheimers Dement 2017; 13: 870–84. 28259709 10.1016/j.jalz.2017.01.014 PMC5788455

[fcaa068-B17] Daianu M, Mezher A, Mendez MF, Jahanshad N, Jimenez EE, Thompson PM. Disrupted rich club network in behavioral variant frontotemporal dementia and early‐onset Alzheimer’s disease. Hum Brain Mapp 2016; 37: 868–83. 26678225 10.1002/hbm.23069 PMC4883024

[fcaa068-B18] Diamond A. Executive functions. Annu Rev Psychol 2013; 64: 135–68. 23020641 10.1146/annurev-psych-113011-143750 PMC4084861

[fcaa068-B19] Dickerson BC, Brickhouse M, McGinnis S, Wolk DA. Alzheimer’s disease: the influence of age on clinical heterogeneity through the human brain connectome. Alzheimers Dement 2017; 6: 122–35. 10.1016/j.dadm.2016.12.007 PMC5318292 28239637

[fcaa068-B20] Dickerson BC, Wolk DA, Alzheimer's Disease Neuroimaging Initiative. Dysexecutive versus amnesic phenotypes of very mild Alzheimer’s disease are associated with distinct clinical, genetic and cortical thinning characteristics. J Neurol Neurosurg Psychiatry 2011; 82: 45–51. 20562467 10.1136/jnnp.2009.199505 PMC3023235

[fcaa068-B21] Dronse J, Fliessbach K, Bischof GN, von Reutern B, Faber J, Hammes J, et al In vivo patterns of tau pathology, amyloid-beta burden, and neuronal dysfunction in clinical variants of Alzheimer’s disease. J Alzheimers Dis 2017; 55: 465–71. 27802224 10.3233/JAD-160316

[fcaa068-B22] Dubois B, Feldman HH, Jacova C, Hampel H, Molinuevo JL, Blennow K, et al Advancing research diagnostic criteria for Alzheimer’s disease: the IWG-2 criteria. Lancet Neurol 2014; 13: 614–29. 24849862 10.1016/S1474-4422(14)70090-0

[fcaa068-B23] Egeland J. Measuring working memory with Digit Span and the Letter-Number Sequencing subtests from the WAIS-IV: too low manipulation load and risk for underestimating modality effects. Appl Neuropsychol Adult 2015; 22: 445–51. 25910198 10.1080/23279095.2014.992069

[fcaa068-B24] Fields JA, Machulda M, Aakre J, Ivnik RJ, Boeve BF, Knopman DS, et al Utility of the DRS for predicting problems in day-to-day functioning. Clin Neuropsychol 2010; 24: 1167–80. 20924981 10.1080/13854046.2010.514865

[fcaa068-B25] Fripp J, Bourgeat P, Acosta O, Raniga P, Modat M, Pike KE, et al Appearance modeling of 11C PiB PET images: characterizing amyloid deposition in Alzheimer’s disease, mild cognitive impairment and healthy aging. Neuroimage 2008; 43: 430–9. 18789389 10.1016/j.neuroimage.2008.07.053

[fcaa068-B26] Gorno-Tempini ML, Hillis AE, Weintraub S, Kertesz A, Mendez M, Cappa SF, et al Classification of primary progressive aphasia and its variants. Neurology 2011; 76: 1006–14. 21325651 10.1212/WNL.0b013e31821103e6 PMC3059138

[fcaa068-B27] Gross RG, Grossman M. Update on apraxia. Curr Neurol Neurosci Rep 2008; 8: 490–6. 18957186 10.1007/s11910-008-0078-y PMC2696397

[fcaa068-B28] Gur RC, Ragland JD, Resnick SM, Skolnick BE, Jaggi J, Muenz L, et al Lateralized increases in cerebral blood flow during performance of verbal and spatial tasks: relationship with performance level. Brain Cogn 1994; 24: 244–58. 8185896 10.1006/brcg.1994.1013

[fcaa068-B29] Hulstaert F, Blennow K, Ivanoiu A, Schoonderwaldt HC, Riemenschneider M, De Deyn PP, et al Improved discrimination of AD patients using beta-amyloid(1-42) and tau levels in CSF. Neurology 1999; 52: 1555–62. 10331678 10.1212/wnl.52.8.1555

[fcaa068-B30] Humpel C. Identifying and validating biomarkers for Alzheimer’s disease. Trends Biotechnol 2011; 29: 26–32. 20971518 10.1016/j.tibtech.2010.09.007 PMC3016495

[fcaa068-B31] Hyman BT, Phelps CH, Beach TG, Bigio EH, Cairns NJ, Carrillo MC, et al National Institute on Aging-Alzheimer’s Association guidelines for the neuropathologic assessment of Alzheimer’s disease. Alzheimers Dement 2012; 8: 1–13. 22265587 10.1016/j.jalz.2011.10.007 PMC3266529

[fcaa068-B32] Ivnik RJ, Smith GE, Lucas JA, Tangalos EG, Kokmen E, Petersen RC. Free and cued selective reminding test: MOANS norms. J Clin Exp Neuropsychol 1997; 19: 676–91. 9408798 10.1080/01688639708403753

[fcaa068-B33] Jack CR, Bennett DA, Blennow K, Carrillo MC, Dunn B, Haeberlein SB, et al NIA-AA Research Framework: toward a biological definition of Alzheimer’s disease. Alzheimers Dement 2018; 14: 535–62. 29653606 10.1016/j.jalz.2018.02.018 PMC5958625

[fcaa068-B34] Jack CR Jr, Bernstein MA, Fox NC, Thompson P, Alexander G, Harvey D, et al The Alzheimer’s Disease Neuroimaging Initiative (ADNI): MRI methods. J Magn Reson Imaging 2008; 27: 685–91. 18302232 10.1002/jmri.21049 PMC2544629

[fcaa068-B35] Jack CR Jr, Knopman DS, Weigand SD, Wiste HJ, Vemuri P, Lowe V, et al An operational approach to National Institute on Aging-Alzheimer’s Association criteria for preclinical Alzheimer disease. Ann Neurol 2012; 71: 765–75. 22488240 10.1002/ana.22628 PMC3586223

[fcaa068-B36] Jack CR Jr, Wiste HJ, Weigand SD, Therneau TM, Lowe VJ, Knopman DS, et al Defining imaging biomarker cut points for brain aging and Alzheimer’s disease. Alzheimers Dement 2017; 13: 205–16. 27697430 10.1016/j.jalz.2016.08.005 PMC5344738

[fcaa068-B37] Johnson JK, Head E, Kim R, Starr A, Cotman CW. Clinical and pathological evidence for a frontal variant of Alzheimer disease. Arch Neurol 1999; 56: 1233–9. 10520939 10.1001/archneur.56.10.1233

[fcaa068-B38] Jones DT, Graff-Radford J, Lowe VJ, Wiste HJ, Gunter JL, Senjem ML, et al Tau, amyloid, and cascading network failure across the Alzheimer’s disease spectrum. Cortex 2017; 97: 143–59. 10.1016/j.cortex.2017.09.018 PMC5773067 29102243

[fcaa068-B39] Jones DT, Vemuri P, Murphy MC, Gunter JL, Senjem ML, Machulda MM, et al Non-stationarity in the “resting brain’s” modular architecture. PLoS One 2012; 7: e39731. 22761880 10.1371/journal.pone.0039731 PMC3386248

[fcaa068-B40] Jurica SJ, Leitten CL, Mattis S. Dementia Rating Scale-2: Professional manual. Odessa, FL: Psychological Assessment Resources; 2001.

[fcaa068-B41] Kaiser NC, Melrose RJ, Liu C, Sultzer DL, Jimenez E, Su M, et al Neuropsychological and neuroimaging markers in early versus late-onset Alzheimer’s disease. Am J Alzheimers Dis Other Demen 2012; 27: 520–9. 22990206 10.1177/1533317512459798 PMC4112191

[fcaa068-B42] Kane MJ, Engle RW. Working-memory capacity and the control of attention: the contributions of goal neglect, response competition, and task set to Stroop interference. J Exp Psychol Gen 2003; 132: 47–70. 12656297 10.1037/0096-3445.132.1.47

[fcaa068-B43] Kaplan E, Goodglass H, Weintraub S. Boston naming test (experimental version) Boston: VA Medical Center; 1976.

[fcaa068-B44] Kokmen E, Smith GE, Petersen RC, Tangalos E, Ivnik RC. The short test of mental status: correlations with standardized psychometric testing. Arch Neurol 1991; 48: 725–8. 1859300 10.1001/archneur.1991.00530190071018

[fcaa068-B45] Kovacs GG, Ferrer I, Grinberg LT, Alafuzoff I, Attems J, Budka H, et al Aging-related tau astrogliopathy (ARTAG): harmonized evaluation strategy. Acta Neuropathol 2016; 131: 87–102. 26659578 10.1007/s00401-015-1509-x PMC4879001

[fcaa068-B46] Kravitz DJ, Saleem KS, Baker CI, Mishkin M. A new neural framework for visuospatial processing. Nat Rev Neurosci 2011; 12: 217–30. 21415848 10.1038/nrn3008 PMC3388718

[fcaa068-B47] Króliczak G, Piper BJ, Frey SH. Specialization of the left supramarginal gyrus for hand-independent praxis representation is not related to hand dominance. Neuropsychologia 2016; 93: 501–12. 27020138 10.1016/j.neuropsychologia.2016.03.023 PMC5036996

[fcaa068-B48] Lohner V, Brookes RL, Hollocks MJ, Morris RG, Markus HS. Apathy, but not depression, is associated with executive dysfunction in cerebral small vessel disease. PLoS One 2017; 12: e0176943. 28493898 10.1371/journal.pone.0176943 PMC5426624

[fcaa068-B49] Lucas JA, Ivnik RJ, Smith GE, Bohac DL, Tangalos EG, Graff-Radford NR, et al Mayo’s older Americans normative studies: category fluency norms. J Clin Exp Neuropsychol 1998; 20: 194–200. 9777473 10.1076/jcen.20.2.194.1173

[fcaa068-B50] Luria A. Higher cortical functions in man New York, NY: Basic Books; 1966.

[fcaa068-B51] Machulda M, Ivnik R, Smith G, Ferman T, Boeve B, Knopman D, et al Mayo’s older Americans normative studies: visual form discrimination and copy trial of the Rey–Osterrieth complex figure. J Clin Exp Neuropsychol 2007; 29: 377–84. 17497561 10.1080/13803390600726803

[fcaa068-B52] Machulda MM, Whitwell JL, Duffy JR, Strand EA, Dean PM, Senjem ML, et al Identification of an atypical variant of logopenic progressive aphasia. Brain Lang 2013; 127: 139–44. 23566690 10.1016/j.bandl.2013.02.007 PMC3725183

[fcaa068-B53] Marek S, Dosenbach N. The frontoparietal network: function, electrophysiology, and importance of individual precision mapping. Dialogues Clin Neurosci 2018; 20: 133–40. 30250390 10.31887/DCNS.2018.20.2/smarek PMC6136121

[fcaa068-B54] Matt E, Foki T, Fischmeister F, Pirker W, Haubenberger D, Rath J, et al Early dysfunctions of fronto-parietal praxis networks in Parkinson’s disease. Brain Imaging Behav 2017; 11: 512–25. 26935551 10.1007/s11682-016-9532-7 PMC5408054

[fcaa068-B55] McDade E, Wang G, Gordon BA, Hassenstab J, Benzinger TL, Buckles V et al for the Dominantly Inherited Alzheimer Network. Longitudinal cognitive and biomarker changes in dominantly inherited Alzheimer disease. Neurology 2018; 91: e1295–306. 30217935 10.1212/WNL.0000000000006277 PMC6177272

[fcaa068-B56] McKhann GM, Knopman DS, Chertkow H, Hyman BT, Jack CR Jr, Kawas CH, et al The diagnosis of dementia due to Alzheimer’s disease: recommendations from the National Institute on Aging-Alzheimer’s Association workgroups on diagnostic guidelines for Alzheimer’s disease. Alzheimers Dement 2011; 7: 263–9. 21514250 10.1016/j.jalz.2011.03.005 PMC3312024

[fcaa068-B57] Mendez MF, Lee AS, Joshi A, Shapira JS. Nonamnestic presentations of early-onset Alzheimer’s disease. Am J Alzheimers Dis Other Demen 2012; 27: 413–20. 22871906 10.1177/1533317512454711 PMC3625669

[fcaa068-B58] Mirra SS, Heyman A, McKeel D, Sumi S, Crain BJ, Brownlee L et al participating CERAD neuropathologists. The Consortium to Establish a Registry for Alzheimer’s Disease (CERAD): part II. Standardization of the neuropathologic assessment of Alzheimer’s disease. Neurology 1991; 41: 479–86. 2011243 10.1212/wnl.41.4.479

[fcaa068-B59] Miyake A, Friedman NP, Emerson MJ, Witzki AH, Howerter A, Wager TD. The unity and diversity of executive functions and their contributions to complex “frontal lobe” tasks: a latent variable analysis. Cogn Psychol 2000; 41: 49–100. 10945922 10.1006/cogp.1999.0734

[fcaa068-B60] Murray ME, Graff-Radford NR, Ross OA, Petersen RC, Duara R, Dickson DW. Neuropathologically defined subtypes of Alzheimer’s disease with distinct clinical characteristics: a retrospective study. Lancet Neurol 2011; 10: 785–96. 21802369 10.1016/S1474-4422(11)70156-9 PMC3175379

[fcaa068-B61] Navon D. Forest before trees: the precedence of global features in visual perception. Cogn Psychol 1977; 9: 353–83.

[fcaa068-B62] Ossenkoppele R, Pijnenburg YA, Perry DC, Cohn-Sheehy BI, Scheltens NM, Vogel JW, et al The behavioural/dysexecutive variant of Alzheimer’s disease: clinical, neuroimaging and pathological features. Brain 2015; 138: 2732–49. 26141491 10.1093/brain/awv191 PMC4623840

[fcaa068-B63] Ossenkoppele R, Schonhaut DR, Scholl M, Lockhart SN, Ayakta N, Baker SL, et al Tau PET patterns mirror clinical and neuroanatomical variability in Alzheimer’s disease. Brain 2016; 139: 1551–67. 26962052 10.1093/brain/aww027 PMC5006248

[fcaa068-B64] Osterrieth P. Filetest de copie d’une figure complexe [The test of a complex copied figure]. Arch Psychol 1944; 30: 206–56.

[fcaa068-B65] Palva JM, Monto S, Kulashekhar S, Palva S. Neuronal synchrony reveals working memory networks and predicts individual memory capacity. Proc Natl Acad Sci U S A 2010; 107: 7580–5. 20368447 10.1073/pnas.0913113107 PMC2867688

[fcaa068-B66] Petersen RC, Smith G, Kokmen E, Ivnik RJ, Tangalos EG. Memory function in normal aging. Neurology 1992; 42: 396–401. 1736173 10.1212/wnl.42.2.396

[fcaa068-B67] Ray MK, Mackay CE, Harmer CJ, Crow TJ. Bilateral generic working memory circuit requires left-lateralized addition for verbal processing. Cereb Cortex 2008; 18: 1421–8. 17947348 10.1093/cercor/bhm175

[fcaa068-B68] Reitan RM. Validity of the Trail Making Test as an indicator of organic brain damage. Percept Mot Skills 1958; 8: 271–6.

[fcaa068-B69] Rey A. L’examen clinique en psychologie [The clinical psychological examination]. Paris: Presses Universitaires de France; 1964.

[fcaa068-B70] Riddoch J, Humphreys G. The Birmingham Object Recognition battery (BORB) Hove, UK: Psychology Press; 1993.

[fcaa068-B71] Rottschy C, Langner R, Dogan I, Reetz K, Laird AR, Schulz JB, et al Modelling neural correlates of working memory: a coordinate-based meta-analysis. Neuroimage 2012; 60: 830–46. 22178808 10.1016/j.neuroimage.2011.11.050 PMC3288533

[fcaa068-B72] Ruff R, Light R, Parker S, Levin H. Benton controlled oral word association test: reliability and updated norms. Arch Clin Neuropsychol 1996; 11: 329–38. 14588937

[fcaa068-B73] Scheltens NM, Tijms BM, Koene T, Barkhof F, Teunissen CE, Wolfsgruber S et al. German Dementia Competence Network. Cognitive subtypes of probable Alzheimer’s disease robustly identified in four cohorts. Alzheimers Dement 2017; 13: 1226–36. 28427934 10.1016/j.jalz.2017.03.002 PMC5857387

[fcaa068-B74] Schwarz CG, Gunter JL, Ward CP, Vemuri P, Senjem ML, Wiste HJ, et al The Mayo Clinic Adult Life Span Template: better quantification across the life span. Alzheimers Dement 2017; 13: P93–4.

[fcaa068-B75] Selemon LD, Goldman-Rakic PS. Common cortical and subcortical targets of the dorsolateral prefrontal and posterior parietal cortices in the rhesus monkey: evidence for a distributed neural network subserving spatially guided behavior. J Neurosci 1988; 8: 4049–68. 2846794 10.1523/JNEUROSCI.08-11-04049.1988 PMC6569486

[fcaa068-B76] Simonsen AH, Herukka SK, Andreasen N, Baldeiras I, Bjerke M, Blennow K, et al Recommendations for CSF AD biomarkers in the diagnostic evaluation of dementia. Alzheimers Dement 2017; 13: 274–84. 28341065 10.1016/j.jalz.2016.09.008

[fcaa068-B77] Smith R, Puschmann A, Schöll M, Ohlsson T, Van Swieten J, Honer M, et al 18F-AV-1451 tau PET imaging correlates strongly with tau neuropathology in MAPT mutation carriers. Brain 2016; 139: 2372–9. 27357347 10.1093/brain/aww163 PMC4995360

[fcaa068-B78] Spreen O, Strauss E, editors. General intellectual ability and assessment of premorbid intelligence. In: A compendium of neuropsychological tests. 1998 p. 43–135.

[fcaa068-B79] Steinberg BA, Bieliauskas LA, Smith GE, Ivnik RJ. Mayo’s older Americans normative studies: age-and IQ-adjusted norms for the trail-making test, the stroop test, and MAE controlled oral word association test. Clin Neuropsychol 2005; 19: 329–77. 16120535 10.1080/13854040590945210

[fcaa068-B80] Stopford CL, Thompson JC, Neary D, Richardson AM, Snowden JS. Working memory, attention, and executive function in Alzheimer’s disease and frontotemporal dementia. Cortex 2012; 48: 429–46. 21237452 10.1016/j.cortex.2010.12.002

[fcaa068-B81] Stroop JR. Studies of interference in serial verbal reactions. J Exp Psychol 1935; 18: 643–62.

[fcaa068-B82] Tang-Wai DF, Knopman DS, Geda YE, Edland SD, Smith GE, Ivnik RJ, et al Comparison of the short test of mental status and the mini-mental state examination in mild cognitive impairment. Arch Neurol 2003; 60: 1777–81. 14676056 10.1001/archneur.60.12.1777

[fcaa068-B83] Thal DR, Rüb U, Orantes M, Braak H. Phases of Aβ-deposition in the human brain and its relevance for the development of AD. Neurology 2002; 58: 1791–800. 12084879 10.1212/wnl.58.12.1791

[fcaa068-B84] Thomas C, Kveraga K, Huberle E, Karnath H-O, Bar M. Enabling global processing in simultanagnosia by psychophysical biasing of visual pathways. Brain 2012; 135: 1578–85. 22418740 10.1093/brain/aws066 PMC3338926

[fcaa068-B85] Townley RA, Syrjanen JA, Botha H, Kremers WK, Aakre JA, Fields JA, et al Comparison of the short test of mental status and the montreal cognitive assessment across the cognitive spectrum. Mayo Clin Proc 2019; 94: 1516–1523. 31280871 10.1016/j.mayocp.2019.01.043 PMC6937135

[fcaa068-B86] Vemuri P, Whitwell JL, Kantarci K, Josephs KA, Parisi JE, Shiung MS, et al Antemortem MRI based STructural Abnormality iNDex (STAND)-scores correlate with postmortem Braak neurofibrillary tangle stage. Neuroimage 2008; 42: 559–67. 18572417 10.1016/j.neuroimage.2008.05.012 PMC3097053

[fcaa068-B87] Walter H, Bretschneider V, Grön G, Zurowski B, Wunderlich AP, Tomczak R, et al Evidence for quantitative domain dominance for verbal and spatial working memory in frontal and parietal cortex. Cortex 2003; 39: 897–911. 14584558 10.1016/s0010-9452(08)70869-4

[fcaa068-B88] Warren JD, Fletcher PD, Golden HL. The paradox of syndromic diversity in Alzheimer disease. Nat Rev Neurol 2012; 8: 451–64. 22801974 10.1038/nrneurol.2012.135

[fcaa068-B89] Wechsler D. The Wechsler Adult Intelligence Scale-Revised (Manual). New York, NY: Psychological Corporation; 1981.

[fcaa068-B90] Wechsler D. Manual for the Wechsler Memory Scale-Revised San Antonio, TX: Psychological Corporation; 1987.

[fcaa068-B91] Wechsler D. WAIS-3: Wechsler Adult Intelligence Scale: Administration and Scoring Manual. San Antonio, TX: Psychological Corporation; 1997.

[fcaa068-B92] Whitwell JL, Jones DT, Duffy JR, Strand EA, Machulda MM, Przybelski SA, et al Working memory and language network dysfunctions in logopenic aphasia: a task-free fMRI comparison with Alzheimer’s dementia. Neurobiol Aging 2015; 36: 1245–52. 25592958 10.1016/j.neurobiolaging.2014.12.013 PMC4346438

[fcaa068-B93] Wickham H. ggplot2: elegant graphics for data analysis New York, NY: Springer; 2009.

[fcaa068-B94] Wolk DA, Dickerson BC, Weiner MAlzheimer's Disease Neuroimaging Initiative. Apolipoprotein E (APOE) genotype has dissociable effects on memory and attentional–executive network function in Alzheimer’s disease. Proc Natl Acad Sci U S A 2010; 107: 10256–61. 20479234 10.1073/pnas.1001412107 PMC2890481

[fcaa068-B95] Zhang Y-J, Xu Y-F, Cook C, Gendron TF, Roettges P, Link CD, et al Aberrant cleavage of TDP-43 enhances aggregation and cellular toxicity. Proc Natl Acad Sci U S A 2009; 106: 7607–12. 19383787 10.1073/pnas.0900688106 PMC2671323

