# Supplementary material for: Progressive dysexecutive syndrome due to Alzheimer’s disease: a description of 55 cases and comparison to other phenotypes
Source: Brain Commun. 2020 May 27;2(1):fcaa068. doi: 10.1093/braincomms/fcaa068 (PMC7325839; doi:10.1093/braincomms/fcaa068)
Supplement: fcaa068_Supplementary_Data [file fcaa068_supplementary_data.docx]

Supplemental Table 1: Time lag between CSF and Tau PET

| **CSF p-tau** | **Tau PET SUVR** | | **Time lag (months)^a^** |
| --- | --- | --- | --- |
| **Figure 1 Participants** | | | |
| 43.3 | 2.77 | 55 | |
| 58.5 | 2.14 | 9 | |
| 60.8 | 2.11 | 5 | |
| 50.1 | 1.86 | 12 | |
| 59.4 | 1.40 | 48 | |
| **Figure 2 Participants with CSF** | | | |
| 125.7 | 3.27 | 24 | |
| 89.7 | 2.88 | 22 | |
| 168.0 | 2.88 | 3 | |
| 109.2 | 2.87 | 20 | |
| 77.0 | 2.78 | 5 | |
| 86.2 | 2.77 | 20 | |
| 82.0 | 2.67 | 12 | |
| 62.9 | 2.01 | 7 | |
| 104.7 | 1.86 | 12 | |
| 72.8 | 1.74 | 8 | |
| 63.0 | 1.40 | 5 | |
| 101.5 | 1.29 | 2 | |
| ^a^Time lag represents number of months CSF was done prior to tau PET. | | | |
